# Demineralized Dentin Matrix Particle-Based Bio-Ink for Patient-Specific Shaped 3D Dental Tissue Regeneration

**DOI:** 10.3390/polym13081294

**Published:** 2021-04-15

**Authors:** Jonghyeuk Han, Wonwoo Jeong, Min-Kyeong Kim, Sang-Hyeon Nam, Eui-Kyun Park, Hyun-Wook Kang

**Affiliations:** 1 Department of Biomedical Engineering, Ulsan National Institute of Science and Technology 50, UNIST-gil, Ulju-gun, Ulsan 44919, Korea; g14619@unist.ac.kr (J.H.); wwjeong@unist.ac.kr (W.J.); minkkim@unist.ac.kr (M.-K.K.); 2 Department of Pathology and Regenerative Medicine, School of Dentistry, Kyungpook National University, 2177 Dalgubeol-daero, Jung-gu, Daegu 41940, Korea; aay0805@naver.com

**Keywords:** demineralized dentin matrix, bio-ink, 3D bioprinting, dental tissue engineering

## Abstract

Demineralized dentin matrix (DDM)-based materials have been actively developed and are well-known for their excellent performance in dental tissue regeneration. However, DDM-based bio-ink suitable for fabrication of engineered dental tissues that are patient-specific in terms of shape and size, has not yet been developed. In this study, we developed a DDM particle-based bio-ink (DDMp bio-ink) with enhanced three-dimensional (3D) printability. The bio-ink was prepared by mixing DDM particles and a fibrinogen–gelatin mixture homogeneously. The effects of DDMp concentration on the 3D printability of the bio-ink and dental cell compatibility were investigated. As the DDMp concentration increased, the viscosity and shear thinning behavior of the bio-ink improved gradually, which led to the improvement of the ink’s 3D printability. The higher the DDMp content, the better were the printing resolution and stacking ability of the 3D printing. The printable minimum line width of 10% *w*/*v* DDMp bio-ink was approximately 252 μm, whereas the fibrinogen–gelatin mixture was approximately 363 μm. The ink’s cytocompatibility test with dental pulp stem cells (DPSCs) exhibited greater than 95% cell viability. In addition, as the DDMp concentration increased, odontogenic differentiation of DPSCs was significantly enhanced. Finally, we demonstrated that cellular constructs with 3D patient-specific shapes and clinically relevant sizes could be fabricated through co-printing of polycaprolactone and DPSC-laden DDMp bio-ink.

## 1. Introduction

In clinic, non-biological dental implants are commonly used to treat tooth loss. However, differences in physiological properties between the dental implants and human dental tissues cause peri-implantitis with alveolar bone loss and periodontal inflammation [1,2]. It has been reported that approximately 64% of donors experience alveolar bone loss within two months after dental implant surgery [3]. Therefore, bioengineered 3D tissue constructs, which use biomaterials and dental cells, have been used to regenerate craniofacial and dental tissues. Many researchers have reported that dental tissue regeneration can be achieved using tissue engineering approaches [4,5,6,7]. However, producing engineered 3D dental constructs with clinically-relevant sizes, patient-specific shapes, and high regenerative capacity is a challenge [7,8,9,10,11,12,13]. In this respect, 3D bioprinting technology has recently garnered the attention of several researchers. The technology can produce 3D freeform cellular constructs using various types of living cells, biomolecules, and biomaterials [14]. It allows the fabrication of patient-specific and macro-sized structures with micro-resolution [15,16]. In addition, biofunctional materials and living cells can be utilized in the technology to improve tissue regeneration [17,18,19,20,21].

Human demineralized dentin matrix (DDM) is a promising biomaterial with excellent bioactive properties that can improve bone and dental tissue regeneration [14,15,16]. DDM, prepared by selectively removing minerals from human dentins, consists of biomimetic chemical components, such as collagen, non-collagen extracellular matrix (ECM) fibers, and various growth factors [17]. In addition, dentin tubules on the DDM surface are known to be effective on the delivery of growth factors [14,18]. Based on an in-vivo study, Li et al. [19] reported that demineralized human dentin can be used to effectively regenerate engineered dentin tissue. Chen et al. [20] showed that a scaffold consisting of an aligned electrospun sheet, demineralized human dentin, and dental pulp tissue-derived extracellular matrix enables the regeneration of dentin-pulp complex and periodontal-like tissue. Thus, many studies have been reported demonstrating the excellence of DDM for dental tissue engineering. However, achieving regeneration of patient-specific shaped dental tissue using conventional approaches can be a complex process because it is difficult to adjust external shape. In this regard, Athirasala et al. [21] introduced a solubilized DDM-based bio-ink to 3D print dental tissues; however, it did not show sufficient printability to produce bioengineered 3D teeth with patient-specific shapes and clinically relevant sizes.

**Figure 1:**
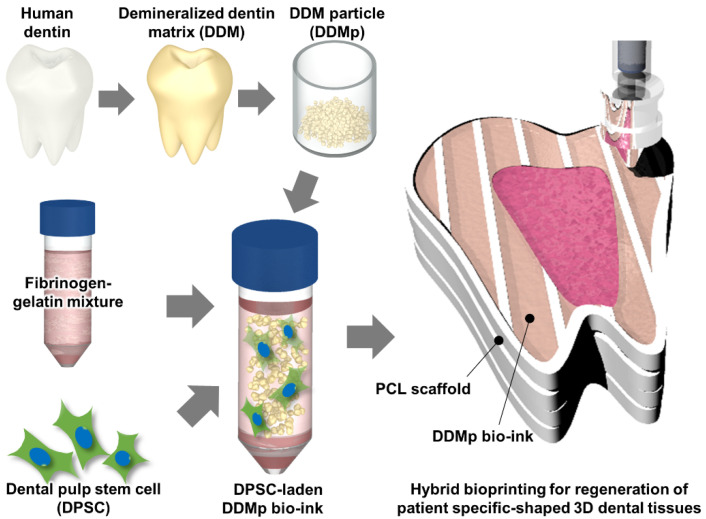
Schematic illustration of the preparation of a demineralized dentin matrix particle (DDMp) bio-ink for 3D bioprinting.

In this study, we developed a new bio-ink for dental tissue engineering using DDM particles (DDMp) (Figure 1) and a fibrinogen–gelatin mixture that is widely used in bioprinting technology. Rheological property, 2D/3D printability, and cytocompatibility of the bio-ink according to the change in DDMp concentration were evaluated and analyzed. Effect of the DDMp-based bio-inks (DDMp bio-inks) on odontogenic differentiation of dental pulp stem cells (DPSCs) was also investigated. Finally, DDMp bio-ink was applied to print a 3D tooth-like cellular construct with patient-specific shape and clinically relevant size using a computer-aided design (CAD) model.

## 2. Materials and Methods

### 2.1. Preparation of DDMp Bio-Ink

DDMp was prepared using human teeth, following the procedure that was reviewed and approved by the Institutional Review Board of Kyungpook National University (Daegu, Korea) (KNU 2017-78). Briefly, the tooth periodontium and pulp were removed, and the dentin was decalcified at 4 °C for 4 weeks with a 0.5 M ethylenediaminetetraacetic acid(EDTA)(Sigma, St. Louis, MO, USA) solution (pH 8.0) containing protease inhibitors (Roche, Basel, Switzerland) as described [22]. Then, the dentin was crushed to make dentin particles [23]. The dentin particles were freeze-milled (Spex Industries, Metuchen, NJ, USA) and sieved by a 100 μm pore-mesh. A fibrinogen–gelatin mixture composed of fibrinogen (Sigma, St. Louis, MO, USA), gelatin (Sigma, St. Louis, MO, USA), hyaluronic acid (HA)(Sigma, St. Louis, MO, USA), and glycerol (Sigma, St. Louis, MO, USA) was prepared based on a previously reported method [24]. Gelatin, HA, and glycerol were used to achieve good printability with living cells. Fibrinogen was used to provide a matrix for cell culturing through crosslinking with thrombin solution, subsequent to printing. To prepare the mixture, HA (3 mg/mL) was dissolved in modified Eagle’s minimum essential medium (MEM; Gibco, Waltham, MA, USA). Gelatin (27.5 mg/mL) and fibrinogen (5 mg/mL) were dissolved into the HA solution (3 mg/mL). Thereafter, glycerol was dissolved to 4% *v*/*v*. The prepared mixture was sterile-filtered (0.45 μm filter; Millipore, Danvers, MA, USA) and stored at –20 °C until used.

Before printing, the frozen mixture was melted in a 37 °C water bath (JEIO tech, Dajeon, Korea). DDMp was sterilized by 70% *v*/*v* ethanol (Samchun chemical, Seoul, Korea) (diluted in distilled water (DW)) for 2 h at 4 °C and washed with DW twice. Subsequently, DDMp was soaked in a growth medium to remove residual ethanol. Finally, the DDMp and the fibrinogen–gelatin mixture were gently mixed to prepare DDMp bio-ink at a concentration of 1–10% *w*/*v*.

### 2.2. Cell Culture and Cell-Laden Bio-Ink Preparation

DPSCs (Lonza, Walkersville, MD, USA) were cultured with alpha MEM (Welgene, Kyungsan, Korea) and supplemented with 10% fetal bovine serum (Capricorn, Hesse, Germany) and 1% *v*/*v* penicillin-streptomycin (P/S; Capricorn, Hesse, Germany) in physiological conditions (37 °C, 5% CO_2_). The growth medium was exchanged every 3 days. DPSCs were subcultured at 70–80% confluency by dissociation with Tryple ™ Select (1X) (Gibco, Waltham, MA, USA). StemMACS OsteoDiff media (Miltenyi Biotec, Bergisch Gladbach, Germany) containing 1% *v*/*v* P/S was used for odontogenic induction of DPSCs. In the culture of DPSC-laden bio-ink, aprotinin (Sigma, St. Louis, MO, USA) was added to the culture medium at a concentration of 10 μg/mL to prevent excessive bio-ink degradation.

Before printing, harvested DPSCs were gently and homogeneously mixed with the prepared DDMp bio-ink (3 × 10^6^ cells/mL). The prepared DPSC-laden-DDMp bio-ink was filled into a 1 mL syringe (Henke Sass Wolf, Nörten-Hardenberg, Germany) and then cooled in a 4 °C refrigerator for 10 min to induce a hydrogel form. Subsequently, the syringe was connected to a micro-nozzle (Mitsumi Electric Co. Ltd., Tokyo, Japan) of 300 μm in diameter and installed into the bioprinting system (Supplementary Materials Figure S1). After the printing process, the DDMp bio-ink was crosslinked with thrombin solution (10 U/mL) (Sigma, St. Louis, MO, USA) for 30–45 min and then cultured for further experiments.

### 2.3. Compressive Modulus and Rheological Property Measurement

Cylindrical samples with a 5 mm diameter and 1 mm height were prepared through the biopsy punching of crosslinked DDMp bio-inks to measure the compressive modulus. After loading each sample into a universal testing machine (Instron Model 3342; Illinois Tool Works Inc., MA, USA), the sample was gradually compressed at a rate of 1 mm/min, and the corresponding force and distance were recorded. After plotting the strain–stress curve, the compressive modulus was measured by calculating a slope based on a 10% strain. In addition, a 3D hybrid construct of 5 × 5 × 5 mm^3^ produced through the co-printing of polycaprolactone (s; MW 43000; Polysciences Inc, Warrington, FL, USA) and DPSCs-laden DDMp bio-ink was measured for the compressive modulus identical to the DDMp bio-ink. The rheological property of the DDMp bio-inks was measured through shear sweep analysis, which was conducted at 0.1–100 s^−1^ using a 20-mm-diameter plate at 18 °C on a HAAKE MARS III Rheometer (Thermo Scientific, Karlsruhe, Germany).

### 2.4. Scanning Electron Microscopy (SEM) Imaging

SEM (S-4800; Hitachi High-Technologies, Tokyo, Japan) was used to investigate the size and morphology of DDM particles. The particles were fixed on a carbon tape and coated with plutonium at 20 mA for 50 s by using a sputter coater (E-1045; Hitachi High-Technologies Co., Tokyo, Japan). SEM images were captured at 5.0 kV, and the particle sizes were measured using the ImageJ software (NIH, Bethesda, MD, USA).

### 2.5. Bioprinting System and Printability Test

A home-made bioprinting system, which can print cell-laden bio-inks, was used to evaluate the DDMp bio-inks (Supplementary Materials Figure S1). The printing system consists of three-axis stages for motion control, multi-cartridge dispensing modules for delivering multiple types of cells and biomaterials, and an enclosure for controlling temperature, humidity, and cleanliness.

The printability of the DDMp bio-inks was evaluated by producing line patterns, and a stacking test of multiple layers was performed to estimate the ink’s 3D printability. The stacking ability of the bio-ink is most evident in stacked line structures such as thin walls. The bio-inks with various concentrations of DDMp were prepared and applied to print line patterns according to the change in printing speed (5–320 mm/min). After imaging the line patterns using a microscope (Leica Dml-1, Leica Microsystems AG, Wetzlar, Germany), their heights and widths were measured using ImageJ software (NIH, Bethesda, MD, USA). Aspect ratios of the line patterns were calculated by dividing height with width. For the stacking test of the DDMp bio-inks, samples were prepared by stacking predefined numbers of line patterns with 400 μm width and 150 μm thickness using the bioprinter. After imaging the printed samples, their stacked heights were measured using the ImageJ software. In the printability tests, DDMp bio-inks were extruded at a rate of 34.3 μL/min through a 300 μm nozzle.

### 2.6. Cytocompatibility

To measure the viability of the DPSCs in DDMp bio-inks and 3D bio-printed constructs, live and dead staining (L3224, Thermo Fisher Scientific, Waltham, MA, USA) was performed on day 7. The samples were stained with assay solution (0.2% *v*/*v* calcein AM (ThermoFisher Scientific, Waltham, MA, USA) and 0.05% *v*/*v* ethidium homodimer-1 (ThermoFisher Scientific, Waltham, MA, USA) in PBS) at room temperature for 1 h and then imaged using a fluorescent microscope (Leica DM2500, Leica Microsystems AG, Wetzlar, Germany). After counting the number of live and dead cells, the cell viability was calculated by dividing the number of live cells by the total cell count.

Proliferation assay was performed using AlamarBlue™ Cell Viability Reagent (ThermoFisher Scientific, Waltham, MA, USA) on days 1, 3, 5, and 7. Bioprinted DPSC-laden bio-inks were incubated in 10% *v*/*v* alamar blue dye diluted by growth medium in an incubator for 3 h. After sampling the 100 μL assay solution to a 96-well plate, their fluorescence intensities (excitation: 544 nm/ emission: 599 nm) were measured using a microplate reader (Synergy NEO2 Hybrid Multi-Mode Reader; Bio-Tek, Winooski, VT, USA). The measured data were normalized by the data of the sample obtained on day 1.

### 2.7. Alizarin Red and Alkaline Phosphate (AP) Staining

Bioprinted DPSC-laden samples were cultured in an odontogenic differentiation medium for 15 days; alizarin red and AP staining were performed. For the alizarin red staining, samples were fixed with 4% formaldehyde solution (Junsei, Tokyo, Japan) solution on day 15 and stained with an alizarin red S (Sigma, St. Louis, MO, USA) solution of 2% *w*/*v* diluted by double DW at pH 4.3 for 20 min. To quantify the mineral deposition, the stained samples were de-stained with 10% cetylpyridinium chloride (Sigma, St. Louis, MO, USA) solution in 10 mM sodium phosphate buffer (Sigma, St. Louis, MO, USA) (pH 7.0) overnight. The optical density of each de-stained sample was measured using a microplate reader at 550 nm. After de-staining, the samples were observed with a microscope. AP staining was performed using REPROCELL Stemgent ® Alkaline Phosphatase (AP) Staining Kit II (Stemgent, Cambridge, MA, USA), according to the manufacturer’s instruction. In particular, the samples were fixed for 2–5 min and stained with the prepared AP staining solution in the dark at room temperature for 5–15 min. Thereafter, the samples were washed twice with PBS. The stained samples were then imaged with a microscope.

### 2.8. Immunofluorescence Staining

Immunostaining for dentin sialo-phosphoprotein (DSPP) was performed. Samples, cultured in the differentiation medium for 15 days, were fixed with 4% PFA for a day. They were permeabilized with 0.1% Triton X-100 (Sigma, St. Louis, MO, USA) in PBS (Sigma, St. Louis, MO, USA) for 5 min and blocked with 5% bovine serum albumin (Sigma, St. Louis, MO, USA) for 1 h. The samples were incubated in the DSPP antibody solution (dilution ratio 1:500; SantaCruz Biotechnology, Dallas, TX, USA) overnight at 4 °C. They were washed with PBS at 4 °C overnight. The secondary antibody, Goat Anti-mouse lgG (H+L) Alexa Fluor 488 (Thermofisher Scientific, Waltham, MA, USA), was diluted in PBS (1:1000) and incubated with the samples for 1 h at room temperature. The samples were washed three times with PBS, followed by incubation in 1:1000 diluted Hoechst 33258 (Sigma, St. Louis, MO, USA) for 3 min. Thereafter, they were observed using a fluorescent microscope.

### 2.9. RT-qPCR

Messenger ribonucleic acid (mRNA) expression levels of dentin matrix acidic phosphoprotein 1 (DMP-1) and DSPP were measured. Each sample was crushed using bioMasher-Ⅱ (Optima, Tokyo, Japan). The samples were treated with Trisure (Bioline, London, UK) for 10 min and vigorously vortexed for 30 s to isolate the RNA. cDNA for each RNA sample was synthesized using helixcript™ Thermo Reversed Transcriptase (Nanohelix, Daejeon, South Korea). Biometra Professional TRIO Thermo-cycler (Analytik Jena AG, Jena, Germany) was used for the synthesis of cDNA. Then, the cDNA mixed with primers (Table S1) was amplified with Roche SYBR Light Cycler 480 SYBR Green I Master (Roche Diagnostics Gmbh, Mannheim, Germany) using a light cycler 480 II (Roche Diagnostics Gmbh, Mannheim, Germany). Expression levels of DMP-1 and DSPP have been normalized to glyceraldehyde 3-phosphate dehydrogenase (GAPDH) via the ΔΔCt method. All the experiments were performed in triplicate.

### 2.10. Bioprinting of 3D Tooth-Shaped Cellular Construct with DDMp Bio-Ink

A tooth-shaped 3D-CAD model was applied to print a patient-specific shaped construct. A motion program was prepared by an in-house computer-aided manufacturing software and the 3D-CAD model [24]. The motion program was loaded into the bioprinter, and 3D cellular constructs were produced through an automated process in which layers of 150 μm thickness were added sequentially in a predefined manner using DPSC-laden DDMp bio-ink (10% *w*/*v* DDMp, 3 × 10^6^ cells/mL) and/or PCL. Bioprinting with DDMp bio-ink was conducted using a 300 μm nozzle at a dispensing rate and printing speed of 0.5735 μL/s and 50 mm/min, respectively. Polymer printing with PCL was conducted using a 300 μm nozzle at a dispensing pressure of 200 Kpa at 85 °C. A bio-ink-only construct has a height of 3 mm and a bio-ink/PCL hybrid construct has a height of 2 cm. In the printing processes, the enclosure was maintained at 18 °C. Subsequently, the construct was crosslinked with thrombin (10 U/mL) solution at room temperature for 45 min and cultured in the incubator.

### 2.11. Statistical Analysis

All variables are expressed as means ± standard error of the mean. Statistical analyses were performed using Origin (2020 version, OriginLab Corp., Northampton, MA, USA). Multiple comparisons between experimental groups were conducted by one-way analysis of variance and Tukey’s multiple comparison test. In all analyses, *p* < 0.05 was taken to indicate statistical significance.

## 3. Results

### 3.1. Preparation and Characterization of DDMp Bio-Ink

**Figure 2:**
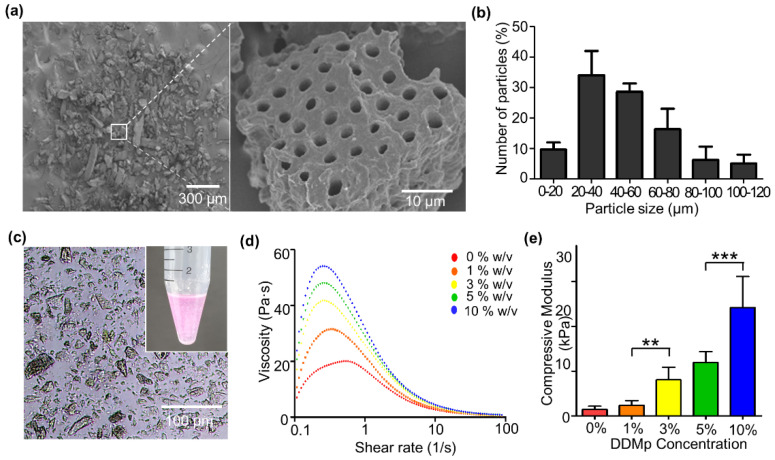
DDMp bio-ink and its rheological property. (**a**) Scanning electron microscopic image of a prepared DDM particles (left) and its magnified (×50) image (right). (**b**) Size distribution of DDM particles (*n* = 3). (**c**) Microscopic image and photograph (inset) of a DDMp bio-ink. (**d**) Measured viscosity and compressive modulus (**e**) of the DDMp bio-inks. ** *p* < 0.01; *** *p* < 0.001.

DDMp was prepared through demineralization and freeze-milling using human teeth. The prepared DDMp had irregular polygonal shapes in micro-scale, and dentinal tubule structures of 2–4 μm diameter were well maintained on the surfaces (Figure 2a). From the measured particle size, the average size of the prepared DDMp was approximately 48.13 μm, of which 20–60 μm sized particles accounted for 62.73% of the total (Figure 2b).

DDMp bio-inks were prepared by mixing the DDMp with the fibrinogen–gelatin mixture. The microscope image showed that the particles in the bio-inks did not form aggregates and were homogeneously distributed (Figure 2c). After preparing DDMp bio-inks of 1%, 3%, 5%, and 10% *w*/*v* concentration, their viscosities were measured. The viscosity of the bio-ink increased with the concentration of DDMp. Additionally, the shear-thinning properties of the bio-inks were reinforced; the viscosity decreased with increasing shear rate (Figure 2d), as the DDMp concentration increased. The DDMp concentration directly affected the mechanical property of the crosslinked bio-ink. Its compressive modulus increased with DDMp concentration (Figure 2e). The modulus of 10% *w*/*v* DDMp bio-ink was approximately 16.3 times higher than that of the fibrinogen–gelatin mixture. Statistical significance was also observed.

### 3.2. Cytocompatibility of DDMp Bio-Ink

**Figure 3:**
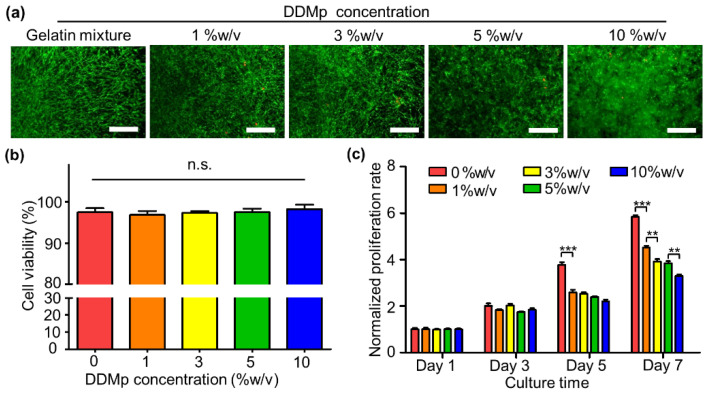
Cytocompatibility of DDMp bio-ink. Live/dead staining results (**a**) and quantified cell viabilities (**b**) of dental pulp stem cells (DPSCs) in DDMp bio-inks at Day 7 (scale bar: 200 μm; *n* = 5); (**c**) proliferation rate of DPSCs in DDMp bio-inks for 7 days (** *p* < 0.01; *** *p* < 0.001, *n* = 4).

DPSCs were applied to investigate the cytocompatibility of the DDMp bio-ink. Live/dead staining results showed that most of the DPSCs were alive in all bio-ink groups regardless of DDMp concentration at day 7 (Figure 3a). Quantification results of the staining indicated greater than 95% cell viability in all groups (Figure 3b). Notably, a significant increase in the DPSC density was observed on and around the DDMp surface in the bio-ink (Supplementary Materials Figure S2). A slightly different trend was observed in the evaluation result of DPSC proliferation, which was performed using an alamar blue assay. All bio-ink groups continued to proliferate for 7 days; however, the DPSC proliferation rate decreased as the DDMp concentration increased (Figure 3c).

### 3.3. Odontogenic Differentiation of DPSCs in DDMp Bio-Ink

**Figure 4:**
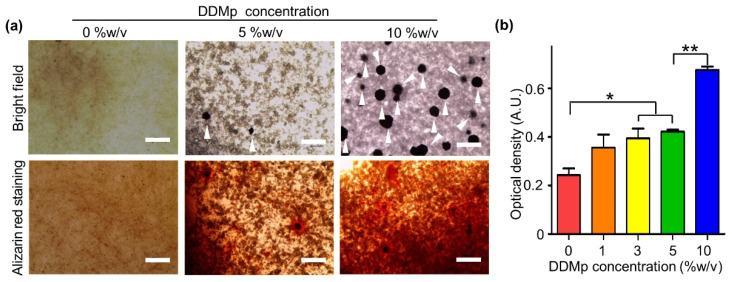
Odontogenic differentiation of DPSC-laden DDMp bio-ink. (**a**) Bright field microscopy images and alizarin red staining result of DPSC-laden DDMp bio-inks after culturing with differentiation medium for 15 days. White arrowheads indicate the formation of mineral nodules (Scale bar: 300 μm). (**b**) Quantification result of alizarin red stained samples (*n* = 4) (* *p* < 0.05; ** *p* < 0.01).

We investigated the effect of DDMp bio-ink on the odontogenic differentiation of DPSCs. After embedding DPSCs in DDMp bio-inks, the samples were cultured in a differentiation medium for 15 days. The microscopic images indicated that the formation of mineral nodules increased with increasing DDMp concentration (upper figures of Figure 4a and Supplementary Materials Figure S3). This tendency was also observed in alizarin red staining results. Mineral deposition increased with increasing DDMp concentration (lower figures of Figure 4a and Supplementary Materials Figure S3). Quantification results with the stained samples showed this trend more clearly (Figure 4b). As the DDMp concentration increased, the optical density increased. The 3%, 5%, and 10% *w*/*v* DDMp bio-ink groups showed significantly higher mineralization than the fibrinogen–gelatin mixture group (0% *w*/*v* concentration).

### 3.4. Printability of DDMp Bio-Ink

**Figure 5:**
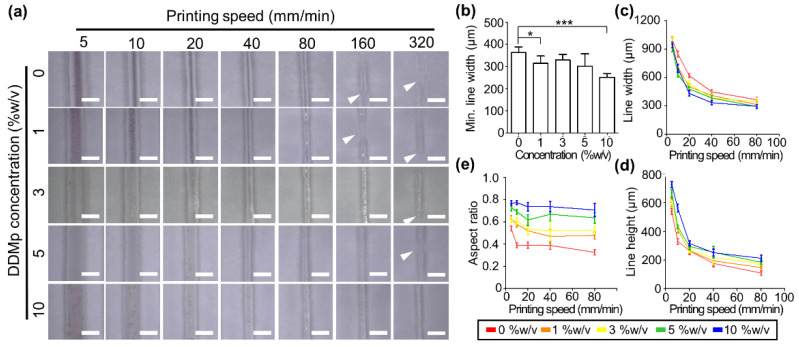
Printability test of DDMp bio-inks. (**a**) Microscopy image of line patterns printed with DDMp bio-inks according to the change of printing speed (scale bar: 1 mm). White arrows indicate disconnected points in the printed lines. Measured results of printable minimum (min.) line width (**b**), line width (* *p* < 0.05; *** *p* < 0.001), (**c**) and height (**d**) of the printed lines (*n* = 10). Min. line width refers to the smallest width among continuous line patterns printed without breaking. (**e**) Aspect ratios of the printed lines, which were calculated using the measured line widths and heights.

We evaluated the 2D and 3D printability of the DDMp bio-inks. For the 2D printability test, line patterns were printed with a 300 μm nozzle and the line width and height were measured. The printed line width gradually decreased as the printing speed increased (Figure 5a). Notably, continuous line patterns were produced more stably as the DDMp concentration increased. The fibrinogen–gelatin mixture group (0% *w*/*v* DDMp) produced broken lines at 160 mm/min printing speed, while the 10% *w*/*v* DDMp bio-ink group produced a continuous line even at 320 mm/min. Based on the continuous line patterns, the fibrinogen–gelatin mixture and 10% *w*/*v* DDMp bio-ink group showed minimum widths of approximately 360 and 252 μm, respectively (Figure 5b). This result indicated that the printing resolution of the 10% *w*/*v* DDMp bio-ink is higher than the other bio-inks. A decreasing trend with increasing printing speed was observed in all groups in the measured line width and height based on the microscope images (Figure 5c,d). As the DDMp concentration increased, the printed line width decreased and the height increased. This tendency was observed more clearly in the calculation result of aspect ratio (Figure 5e). Based on the 320 mm/min printing speed, the 10% DDMp bio-ink and fibrinogen–gelatin mixture group had an aspect ratio of 0.707 and 0.326, respectively.

**Figure 6:**
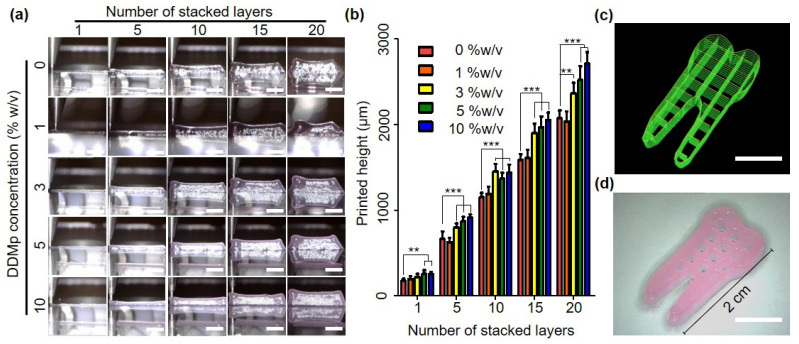
Stacking test of DDMp bio-inks. Microscopy images (scale bar: 5 mm) (**a**) and measured heights (**b**) of multilayered structures printed with DDMp bio-inks in the change of the number of stacked layers (*n* = 6), (**c**) visualized printing code and (**d**) fabrication result of tooth-shaped hydrogel construct printed with DPSC-laden DDMp bio-ink (10% *w*/*v*) (scale bar: 10 mm). The visualized code indicates the printing paths to produce the designed 3D structure. ** *p* < 0.01; *** *p* < 0.001.

In the stacking test of the line patterns, a similar trend was observed with the 2D printing test results. The layer-by-layer process was conducted with a line pattern of 400 μm width and 150 μm thickness. The stacked thickness continued to increase as the number of layers stacked increased in all groups (Figure 6a). Furthermore, DDMp affected the stacked thickness. The thickness increased as the DDMp concentration increased. The measurement results of the thickness based on the microscopy images show this trend more clearly (Figure 6b). There was no significant difference between the fibrinogen–gelatin mixture and the 1% *w*/*v* DDMp bio-ink group; however, for above 3% *w*/*v*, the stacked height significantly increased as the DDMp concentration increased. Finally, the 10% DDMp bio-ink was applied to produce multilayered hydrogel structures. The layer-by-layer process was conducted using the tooth-shaped porous pattern of 2 cm height and 150 μm thickness. The printed hydrogel structure retained the pores suitably without collapse, even for the stacking process involving 20 layers (Figure 6c).

### 3.5. Bioprinting of Human Tooth-Shaped 3D Construct with DPSCs

**Figure 7:**
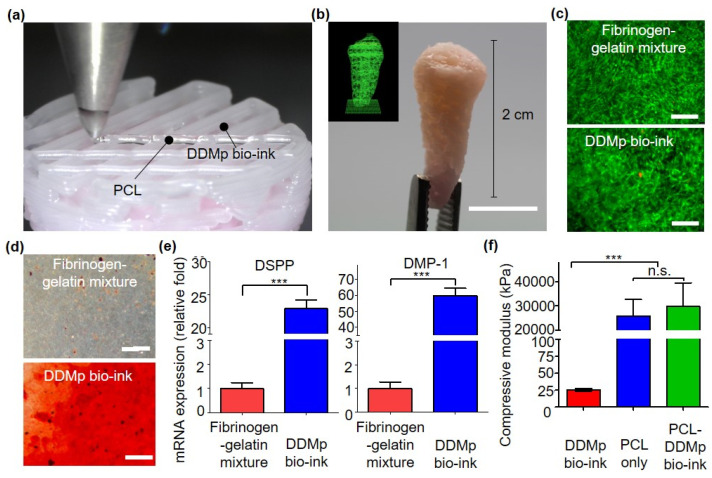
The 3D hybrid bioprinting of human tooth-shaped construct with DPSC-laden DDMp bio-ink and polycaprolactone (PCL) (**a**) 3D hybrid bioprinting procedure (**b**) visualized printing code (inset) and its fabrication result of 3D human tooth-shaped construct with the DPSC-laden bio-ink and PCL (scale bar: 10 mm). (**c**) Live/dead staining results of bioprinted DPSC-laden fibrinogen–gelatin mixture and 10% *w*/*v* DDMp bio-ink at day 7 (Scale bar: 500 μm). Alizarin red staining results (**d**) and relative mRNA expression levels (Dentin Sialo-phosphoprotein (DSPP) and Dentin matrix protein-1 (DMP1) (scale bar: 200 μm)) (**e**) of the constructs printed with DPSCs-laden DDMp bio-ink (10% *w*/*v*) after culturing with odontogenic differentiation medium for 15 days (*** *p* < 0.001, *n* = 3, all the experiments were performed in triplicate). (**f**) Compressive modulus of 3D-printed DDMp bio-ink only, PCL only and PCL/DDMp bio-ink constructs.

We investigated that 3D tooth-shaped cellular constructs can be fabricated through co-printing of DPSC-laden DDMp bio-ink and PCL. A motion program required for the printing process was prepared using a tooth CAD model and home-made software [24]. A 3D tooth-shaped scaffold of 2 cm height was produced through PCL printing, and a DPSC-laden DDMp bio-ink was printed inside it (Figure 7a,b). The pattern of the DDMp bio-ink printed inside the PCL scaffold was stably maintained. The viability and odontogenic differentiation of DPSCs within the printed construct were evaluated. Construct printed with DPSC-laden fibrinogen–gelatin mixture was used as a control group. Owing to live/dead staining after culturing in growth medium for 7 days, most DPSCs were alive in both the groups (fibrinogen–gelatin mixture and DDMp bio-ink group) (Figure 7c). This result is consistent with that depicted in Figure 3b. After culturing in a differentiation medium for 15 days, odontogenic differentiation of the printed tooth-shape construct was evaluated. Alizarin red staining results showed that mineralization was significantly higher in the DDMp bio-ink group than in the fibrinogen–gelatin mixture group (Figure 7d). This result is consistent with that shown in Figure 4. DSPP and DMP-1 expression levels, which are specific markers of odontogenic differentiation, were also measured through RT-qPCR. The group printed with the DDMp bio-ink showed 22.86 and 59.76 times higher DSPP and DMP-1 expression than the fibrinogen–gelatin mixture group, respectively (Figure 7e). Statistical significance was also observed. Furthermore, immunostaining results indicated that AP and DSPP expression was higher around the DDM particle in the bio-ink (Supplementary Materials Figure S4). Finally, the compressive modulus of the cellular construct was measured. A 5 × 5 × 5 mm^3^ cube with an identical inner architecture as the tooth-shaped construct was prepared for compression testing. The measurement results indicated that the PCL-DDMp bio-ink hybrid construct possessed a compressive modulus of approximately 30 MPa, similar to the PCL scaffold (Figure 7f). Alternatively, the hydrogel construct composed of the DDMp bio-ink possessed a modulus of approximately 25 KPa, which was 1165 times lower than that of the hybrid construct. These results indicated that the mechanical property of the 3D tooth-shaped cell structure was mainly determined by the PCL structure.

## 4. Discussion

In dental tissue engineering, the development of engineered dental tissue with patient-specific shape and clinically relevant size is an important issue [25,26,27,28]. In this regard, various studies have been conducted to apply bioprinting technology, capable of producing 3D freeform structure using various cells and biomaterials, in dental tissue engineering [29]. DDM is one of the most promising biomaterials in dental tissue engineering because of its excellent performance in dental tissue regeneration [16,18,19,20,21,23,30]. Various DDM-based materials have been developed. However, DDM-based bio-ink possessing sufficient printability to produce patient-specific shapes and sizes for dental tissue engineering has not been developed yet. In this study, we developed a novel DDM particle-based bio-ink (DDMp bio-ink) that exhibits not only good performance in dental tissue regeneration but also excellent printability.

The fabricated DDMp bio-ink significantly promoted odontogenic differentiation of dental cells. Therefore, the effect of the bio-ink on odontogenic differentiation of DPSCs was investigated. The results indicated that the DDMp bio-ink enhanced the DSPP, DMP-1, and AP expressions and mineralization of DPSCs, which are known as markers of odontogenic differentiation (Figure 4 and Figure 7d–e, Supplementary Materials Figures S3 and S4) [31,32,33,34,35,36]. DPSCs in 10% *w*/*v* DDMp bio-ink possessed polygonal morphologies and their proliferation rate decreased with increasing DDMp concentration (Figure 3a,c and Figure 7c). This phenomenon was observed during odontogenic differentiation of DPSCs. Similar to results we achieved, Al-Sharabi et al. [37] reported that human dental pulp cells possessed polygonal morphologies with reduced proliferation rate in an odontogenic induction medium. Human tooth-derived DDM is known to contain various biochemical factors such as BMP-2, TGF-ß, and bFGF [14,16,18,22,23,38]. It is interpreted that the biochemical factors possessed by DDM particles in the bio-ink promoted the odontogenic differentiation of DPSCs.

The DDM particles enhanced the 3D printability of the bio-ink. DDM particles improved the bioprinting resolution of the fibrinogen–gelatin mixture. As the DDM particle content in the bio-ink increased, printing a smaller line width became possible. The minimum line width that could be produced with 10% *w*/*v* DDMp bio-ink was approximately 252 μm, while the fibrinogen–gelatin mixture was approximately 363 μm (Figure 5b). In addition, DDM particles improved the stacking ability of the bio-ink. Our DDMp bio-ink was able to stack more than 20 layers with line patterns without any additional supporting material (Figure 6a,b). Conversely, DDM-based bio-inks introduced by other researchers exhibited only four layers of stacking results [39]. This stacking ability has a great influence on reliably performing layer-by-layer process for 3D printing. The improved resolution and stacking ability of the DDMp bio-ink is because the DDM particles reinforced the viscosity and shear-thinning behavior of the bio-ink (Figure 2d) [40]. Shear-thinning behavior refers to the phenomenon in which the viscosity increases as the shear rate decreases. This property is one of the most important properties of bio-ink in that it reduces the shear stress acting on cells during the extrusion process for bioprinting and improves shape retention of bioprinted structures [41,42,43].

Finally, we demonstrated that the possibility of fabricating a 3D cellular construct with patient-specific shape and size through co-printing of DDMp bio-ink and PCL (Figure 7a,b). Within the printed construct, DPSCs showed high viability for seven days, and enhanced odontogenic differentiation was also observed (Figure 7d–f). This result indicates that PCL printed at 85 °C did not cause significant thermal damage to DPSCs. This is because the PCL material was printed into a microstructure and subsequently was rapidly cooled down in an environment with a room temperature of 18 °C. These results are consistent with previous cases of co-printing of cell-laden bio-ink and PCL [24,44,45,46]. Our DDMp bio-ink/PCL hybrid construct exhibited a compressive modulus of approximately 29.8 MPa, which is lower than that of native dentin tissue [47]. The hybrid structure exhibited a compressive modulus similar to that of the PCL-only construct, which means that the mechanical properties of the hybrid structure mainly depended on the PCL frame (Figure 7f). In our future study, we plan to develop a co-printing process of DDMp bio-ink and high-strength material, such as ceramics, to enhance mechanical properties of the engineered dental tissues. Moreover, we plan to verify the feasibility and efficacy of the bioprinted 3D dental construct in dental tissue regeneration through in vivo studies.

## 5. Conclusions

In this study, a new DDMp bio-ink was developed for dental tissue engineering by mixing human tooth-derived DDMp with a fibrinogen–gelatin mixture. The DDMp bio-ink could be applied to produce 3D structures through a layer-by-layer process with 2D micro-patterning using living dental cells. The DDMp bio-ink significantly improved in-vitro odontogenic differentiation of DPSCs. A CAD model-based 3D cellular construct of 2 cm height was produced using the proposed bio-ink. This demonstrated that the proposed bio-ink can be applied to bioprint-engineered 3D dental constructs with patient-specific shapes and clinically relevant size. The proposed technology can be regarded as a useful tool in dental tissue engineering.

## Data Availability

The data presented in this study are available on request from the corresponding authors.

## Supplementary Materials

The following are available online at https://www.mdpi.com/article/10.3390/polym13081294/s1. Supplementary Materials Figure S1: Schematic diagram and photograph of a home-made 3D bioprinter, Figure S2: Fluorescence images of DPSCs in 10% *w*/*v* DDMp bio-ink on days 1, 5, and 10, Supplementary Materials Figures S3–S4: Odontogenic differentiation of DPSCs in DDMp bio-ink. Supplementary Materials Table S1: Primer sequences.
