# Supplementary material for: Demineralized Dentin Matrix Particle-Based Bio-Ink for Patient-Specific Shaped 3D Dental Tissue Regeneration
Source: Polymers (Basel). 2021 Apr 15;13(8):1294. doi: 10.3390/polym13081294 (PMC8071469; doi:10.3390/polym13081294)
Supplement: Supplementary file 1 [file polymers-13-01294-s001.zip › polymers-1141116-supplementary.pdf]

Supporting Information

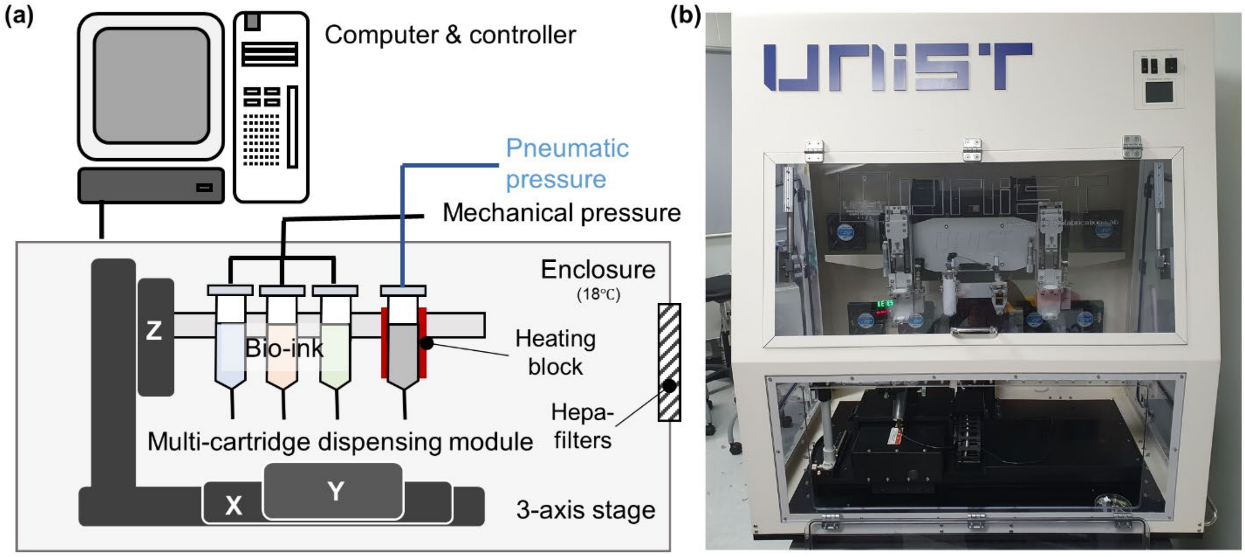

Figure S1. (**a**) Schematic diagram and (**b**) photograph of a home-made 3D bioprinter.

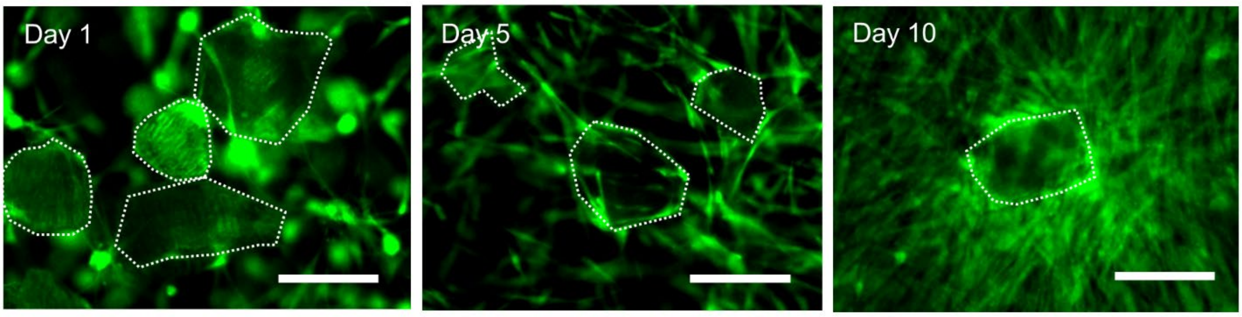

Figure S2. Fluorescence images of DPSCs in 10 %w/v DDMp bio-ink on days 1, 5, and 10 (Scale bars: 20 μm). DPSCs-laden DDMp bio-inks were cultured in proliferation medium for 10 days, and the samples were stained with calcein AM. White dot lines represent DDMp.

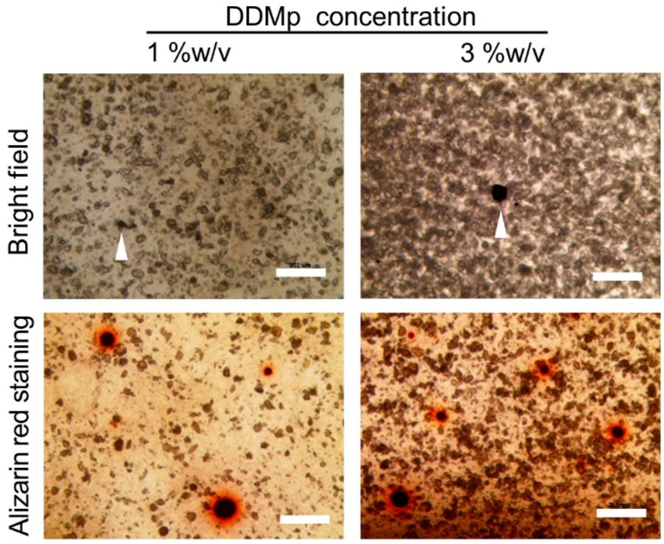

Figure S3. Odontogenic differentiation of DPSCs in DDMp bio-ink. Bright field microscopy images and alizarin red staining results of DPSCs-laden DDMp bio-inks after culturing with differentiation medium for 15 days. White arrowheads indicate the formation of mineral nodules (Scale bar: 500 μm).

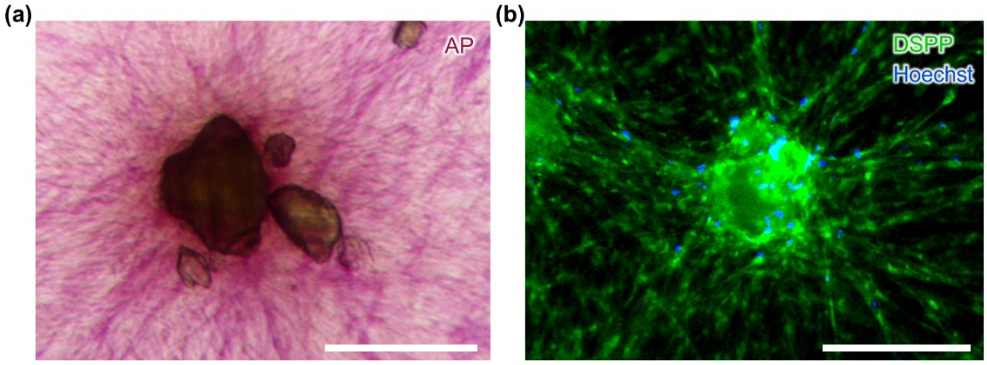

Figure S4. Odontogenic protein expression of DPSCs in 10 %w/v DDMp bio-ink. (**a**) Akaline phosphatase (AP; dark bluish–purple) staining and (**b**) dentin sialophosphoprotein (DSPP; green) immunofluorescence staining results of DPSCs-laden DDMp bio-ink after culturing in odontogenic differentiation for 15 days. Cell nuclei were stained with Hoechst (blue). (Scale bars: 100 μm).

Table S1. Genes and their primer sequences for RT-qPCR.

**Genes**	Primer Sequences	
**GAPDH**	Forward (5′ → 3′)	GAA-GGT-GAA-GGT-CGG-AGT
	Reverse (3′ → 5′)	GAA-GAT-GGT-GAT-GGG-ATT-TC
DMP-1	Forward (5′ → 3′)	CAA-GAC-AGT-GCC-CAA-GAT-AC
	Reverse (3′ → 5′)	TTC-CCT-CAT-CGT-CCA-ACT
DSPP	Forward (5′ → 3′)	CTG-GTG-CAT-GAA-GGT-GAT-AG
	Reverse (3′ → 5′)	CCC-TCT-TCG-TTT-GCT-AAT-GT
